# Do polyethylene wear particles affect the development of pseudotumor in total hip arthroplasty? A minimum 15-year follow-up

**DOI:** 10.1186/s13018-023-03634-7

**Published:** 2023-02-28

**Authors:** Tsunehito Ishida, Toshiyuki Tateiwa, Yasuhito Takahashi, Toshinori Masaoka, Takaaki Shishido, Kengo Yamamoto

**Affiliations:** 1 Department of Orthopedic Surgery, Tokyo Medical University, 6-7-1 Nishishinjuku, Shinjuku-Ku, Tokyo, 160-0023 Japan; 2 Department of Bone and Joint Biomaterial Research, Tokyo Medical University, Tokyo, Japan

**Keywords:** Adverse local tissue reaction, ALTR, Pseudotumor, Total hip arthroplasty, Polyethylene wear, Metal-on-polyethylene, Ceramic-on-polyethylene

## Abstract

**Background:**

Adverse local tissue reactions have been problematic as an implant-related complication in total hip arthroplasty (THA). Despite the absence of significant metal wear and corrosion, granulomatous pseudotumor has been reported to be caused by polyethylene wear. We performed a long-term follow-up study investigating the relationship between polyethylene wear and pseudotumor formation in THA.

**Methods:**

This study included 57 patients (64 hips) that underwent primary THA with metal-on-polyethylene or ceramic-on-polyethylene bearing over a minimum follow-up of 15 years. They were stratified into pseudotumor and non-pseudotumor groups and their linear wear rates of polyethylene liner and serum cobalt (Co) and chromium (Cr) ion levels were compared. Pseudotumor was diagnosed on metal artifact reduction sequence-MRI according to its composition and wall thickness using the Hauptfleisch classification.

**Results:**

The incidence of pseudotumor was 34% (22/64 hips) at the mean follow-up of 16.9 years. Metal ion levels did not differ between the pseudotumor and non-pseudotumor groups, and none of the patients exceeded the Co/Cr ratio of 2.0 μg/L. Moreover, the wear rate in the pseudotumor group was 1.8 times greater than in the non-pseudotumor group (0.14 vs. 0.08 mm/year, *P* < 0.001). According to an analysis of the receiver operating characteristic curves, the cutoff level of the wear rate to discriminate between pseudotumor and non-pseudotumor patients at 15 years was 0.11 mm/year (area under the curve = 91%; sensitivity = 95%; specificity = 78%; accuracy = 87%).

**Conclusions:**

Our results might provide new insights into excessive polyethylene wear potentially leading to the future development of both pseudotumor and osteolysis. Further studies are needed to clarify the direct relationship between polyethylene wear and pseudotumor and the mutual effects of osteolysis and pseudotumor in particle reactions.

## Introduction

Periprosthetic osteolysis and aseptic loosening have been one of the most common reasons for revision in total hip arthroplasty (THA), which commonly proceed via foreign body reactions to wear particles of ultra-high molecular weight polyethylene (UHMWPE; henceforth referred to as polyethylene) bearing [1]. Their risk would increase in vivo as wear particles accumulated, and a linear wear rate of polyethylene greater than 0.1 mm/year generally indicates a radiologic osteolysis threshold [2, 3].

On the other hand, adverse local tissue reactions (ALTR) and pseudotumor formation are also problematic as implant-related complications in THA. In recent publications, ALTR has been associated with mechanical wear and corrosion of metallic components at the modular head-stem taper junction or related to modular stems [4, 5]. However, granulomatous pseudotumor was occasionally observed in the absence of significant metal wear and corrosion, and very few researchers discussed this phenomenon in relation to foreign body reactions to polyethylene wear particles [6–8]. An early study performed by Austin and Stoney [9] suggested that excessive polyethylene wear can result in granulomatosis in patients who have metal-on-polyethylene (MoP) bearings. In one animal-based study, Howie et al. [10] noted that the multinucleate giant-cell response occurred in response to large polyethylene particles and aggregates of small polyethylene particles, implying the presence of ALTR around joint replacements having a polyethylene component. Nevertheless, the relationship between ALTR and polyethylene wear has yet to be fully established.

In the above context, we designed the long-term follow-up study to investigate the following: (1) the incidence of pseudotumor formation after primary THA using polyethylene; and (2) the best cutoff threshold of the wear rate in differentiating between pseudotumor and non-pseudotumor patients. This is the first report on the relationship between polyethylene wear and pseudotumor formation in THA.

## Materials and methods

### Patients

The institutional review board of our hospital approved this retrospective cohort study. Between April 1998 and December 2003, primary cementless THAs were performed for 89 patients (98 hips) at a single institution. All patients received a cementless plasma-sprayed porous-coated RingLoc® acetabular shell, a 30-kGy cross-linked ArCom® polyethylene liner, a 28 mm cobalt chromium (CoCr) or zirconia femoral head and a single design of proximally porous-coated Bi-Metric® femoral stem. Zimmer-Biomet, Warsaw, IN (formerly known as Biomet Inc.) supplied all components. All operations were performed by three experienced hip surgeons (T.S., T.M. and K.Y.) via posterior approach under general anesthesia. Among the 89 patients, 6 patients (6 hips) died for reasons unrelated to THA. The analysis excluded 8 patients (10 hips) who had been followed for less than 15 years. Moreover, 4 patients (4 hips) with rheumatoid arthritis and 14 patients (14 hips) with incomplete MRI data were excluded. A total of 57 patients (64 hips) were included in the analysis and were followed up for a mean of 18.5 years. Demographic data and clinical information are shown in Table 1.

**Table 1:** Characteristics of patients with and without pseudotumor

Variable	Overall (n = 64)	Non-pseudotumor (n = 42)	Pseudotumor (n = 22)	*P* value	Effect size
Age at surgery*	58.0 ± 9.8	60.2 ± 10.0	54.0 ± 8.2	**0.029**	0.29^†^
Gender (female: male)	54: 10	37: 5	17: 5	0.292	n/a
Diagnosis (OA: ION)	58: 6	40: 2	18: 4	0.169	n/a
BMI (kg/m^2^)*	22.3 ± 3.0	22.7 ± 2.9	21.6 ± 2.9	0.109	n/a
Preoperative hip extension limitation (+ : -)^†††^	(16: 48)	(7: 35)	(9: 13)	**0.033**	0.27^††^
Head material (metal: ceramic)	34: 30	22: 20	12: 10	> 0.999	n/a
Cup diameter (mm)*	50.9 ± 2.5	50.8 ± 2.5	51.2 ± 2.5	0.620	n/a
Polyethylene liner thickness (mm)*	6.9 ± 1.3	6.8 ± 1.3	7.0 ± 1.4	0.471	n/a
HHS (points)*	87.2 ± 5.9	89.6 ± 5.8	85.4 ± 6.7	0.184	n/a
Cup inclination (°) at 5y*	42.9 ± 6.0	43.7 ± 5.9	41.4 ± 6.1	0.242	n/a
Cup inclination (°) at 15y*	45.4 ± 5.8	45.7 ± 5.9	44.7 ± 5.4	0.625	n/a
Change in cup inclination between 5 and 15y (°)*	2.4 ± 2.1	2.0 ± 1.6	3.3 ± 2.6	**0.025**	0.28^†^
Cup anteversion (°) at 5y*	16.6 ± 10.3	15.9 ± 10.7	18.0 ± 9.4	0.466	n/a
Cup anteversion (°) at 15y*	20.2 ± 10.8	18.7 ± 11.2	23.4 ± 9.0	0.096	n/a
Change in cup anteversion between 5 and 15y (°)*	3.7 ± 2.6	2.9 ± 1.9	5.4 ± 2.8	**0.001**	0.43^†^
PT (°) at 5 y*	10.9 ± 6.5	10.4 ± 7.3	11.9 ± 4.5	0.336	n/a
PT (°) at 15 y*	16.1 ± 6.9	14.5 ± 6.4	19.0 ± 6.7	**0.041**	0.26^†^
Change in PT between 5 and 15y (°)**	5.2 ± 3.8	4.1 ± 2.9	7.2 ± 4.5	**0.008**	0.33^†^
Frequency of osteolysis (cup)	15 (23%)	6 (14%)	9 (41%)	**0.028**	0.33^††^
Frequency of osteolysis (stem)	14 (22%)	6 (14%)	8 (36%)	0.058	n/a
Cobalt ion level (μg/L)**	0.2 (0.2–0.3)	0.2 (0.2–0.3)	0.3 (0.2–0.5)	0.200	n/a
Chromium ion level (μg/L)**	0.3 (0.2–0.4)	0.3 (0.2–0.4)	0.4 (0.3–0.5)	0.259	n/a
Cobalt chrome ratio**	1.0 (0.6–1.2)	1.0 (0.5–1.0)	1.0 (0.8–1.3)	0.248	n/a

*OA* osteoarthritis; *ION* idiopathic osteonecrosis of the femoral head; *BMI* body mass index; *HHS* Harris hip score; *PT* pelvic tilt; *n/a* not available

^*^Values are presented as the mean and the standard deviation

**Values are presented as the median and the interquartile range

^†^*r*

^††^Cramer’s *V*

^†††^Extension restriction-positive indicates a preoperatively measured hip extension of < 0°, while extension restriction-negative indicates that of ≥ 0°. Numbers in bold are statistically significant

### Clinical and radiographic outcomes

Harris hip scores (HHS) were used to assess clinical outcomes before and after THA. Standard anteroposterior (AP) pelvic and lateral radiographs of hips were obtained at the follow-up to evaluate periprosthetic osteolysis and aseptic loosening of femoral stems and acetabular cups in each of the 7 Gruen zones [11] and the 3 DeLee and Charnley zones [12]. According to the method described by Zicat et al. [13], periprosthetic osteolysis was defined as a focal area of bone resorption that was ≥ 2 mm wide, that was not evident on the immediate postoperative radiograph. Acetabular component with progressive migration of ≥ 3 mm was considered loosening [14]. We evaluated the postoperative pelvic tilt (PT) at 5 and 15 years using a formula based on the sacro-femoral-pubic (SFP) angle measured on frontal radiographs: PT = 75–SFP angle [15].

### Wear analysis

Two-dimensional polyethylene liner wear (femoral head penetration) and acetabular cup inclination and anteversion angle were measured on AP-pelvic radiographs taken in the supine position using Martell’s Hip Analysis Suite software (version 8.0. 4.5; University of Chicago, IL), which is a computer-assisted semiautomated edge detection system. Liner head penetration rates (mm/year [y]) were measured radiographically for all THAs at 5, 10 and 15 years after surgery. Annual radiographs after revision surgery were excluded. All radiographic measurements were analyzed by the same independent observer (T.I.).

### MARS-MRI and metal ion analysis

Metal artifact reduction sequence magnetic resonance imaging (MARS-MRI) was performed after a minimum of 15 years using a 1.5-T scanner (MAGNETOM Avanto fit; Siemens Healthineers, Erlangen, Germany) to diagnose a periprosthetic pseudotumor formation regardless of patients’ symptoms. All MARS-MRIs were independently reviewed by a senior radiologist familiar with pseudotumor diagnosis. Pseudotumors were classified according to their composition and wall thickness using the Hauptfleisch classification [16]: Type I, thin-walled (< 3 mm) cystic masses; Type II, thick-walled cystic masses (> 3 mm but less than the diameter of the cystic component); and Type III, predominantly solid masses. The volume of the lesion was calculated using the formula for the volume of an ellipsoid (volume = 0.52 × height × width × depth) [17].

Serum metal (cobalt [Co] and chromium [Cr]) ion levels and the Co/Cr ratio were measured 1 year after MARS-MRI. Serum Co levels in patients were measured using inductively coupled plasma mass spectrometry at Mayo Medical Laboratories (Rochester, MN, USA), while Cr levels were using atomic absorption spectrometry at LSI Medience Corporation (Tokyo, Japan). Both Co and Cr ions had detection limits of 0.1 μg/L.

### Statistical analysis

The Mann–Whitney's *U* test and the *χ*^2^ test were used to compare the patient groups with and without pseudotumor. The *r* and Cramer’s* V* were reported as indicators of effect sizes for the Mann–Whitney *U* and *χ*^*2*^ test, respectively, considering that *r* and *V* = 0.1 represent a small effect, *r* and *V* = 0.3 represent a medium effect, and *r* and *V* = 0.5 represent a large effect [18, 19]. To determine the ability to distinguish the patient groups, a receiver operating characteristic (ROC) analysis was performed on the wear rates determined at 5, 10 and 15 years postoperatively. The optimal cutoff value of the wear rate was determined to maximise sensitivity, and its overall accuracy was measured by the area under the ROC curve (AUC) as the ability to predict the presence of pseudotumor. The sensitivity (true positive rate), specificity (false positive rate), and overall accuracy (defined as the average of sensitivity and specificity) were also determined by the results of the above discrimination. All statistical analyses were performed using the GraphPad Prism software, version 8.4.1 (GraphPad Software Inc., San Diego, CA, USA). In addition, a *P* value of < 0.05 was considered statistically significant.

## Results

### Clinical and radiographic outcomes

We reviewed the clinical and radiographic outcomes of 57 patients (64 hips) who had MoP (34 hips) and ceramic-on-polyethylene (CoP: 30 hips) bearings. The mean follow-up was 18.5 years (range: 15–23 years). At the time of final follow-up, the mean (standard deviation, SD) HHS had increased significantly from 43.3 (4.7) to 87.2 (5.9) (*P* < 0.001). Acetabular and femoral osteolysis were observed in 15 hips (23%) and 14 hips (22%), respectively (Table 1). They were observed in the DeLee zones 1 (3hips) and 2 (6hips) and both zones (5hips) on the acetabular side, while they were in the Gruen zones 1 (10hips) and 7 (1hip) and both zones (3hips) on the femoral side. However, there was no radiographic loosening of the prosthesis at the final follow-up. The median (interquartile, IQR) PT angle increased from 11° (6–15) at 5 years to 15° (11–20.5) at 15 years postoperatively.

### Comparisons among patients with and without pseudotumor

**Fig. 1:**
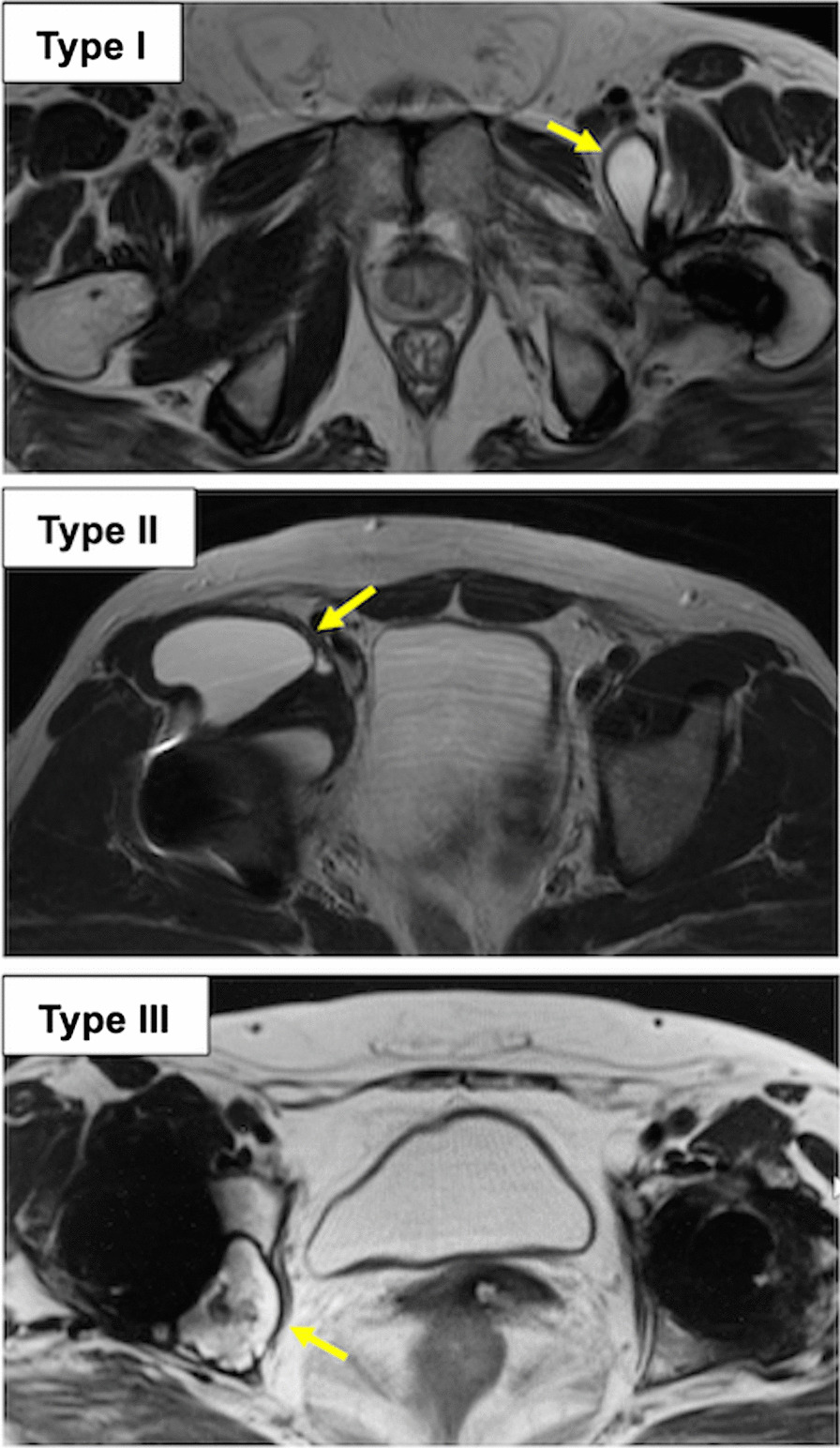
Representative metal artefact reduction sequence magnetic resonance imaging (MARS-MRI) findings of pseudotumor (arrows) classified as Hauptfleisch’s types I, II and III, respectively

The mean (SD) time elapsed between the primary THA and MARS-MRI scanning was 16.9 (1.8) years. MARS-MRI revealed the presence of pseudotumor formation in 22 hips (34%). The pseudotumor was classified as type I (18 hips, 82%), type II (2 hips, 9%), and type III (2 hips, 9%) (Table 2). Figure 1 depicts representative images of types I–III pseudotumor. Most of the lesions were relatively small, with a median (IQR) volume of 3.7 cm^3^ (1.3–9.8). Moreover, five of 22 hips (23%) were symptomatic, with a median (IQR) volume of 11.4 cm^3^ (3.5–15.9), and 4 of the symptomatic 5 hips underwent revision surgery after a mean (SD) follow-up period of 12.4 (2.5) years (Table 2). According to the Musculoskeletal Infection Society criteria, none of them had periprosthetic joint infection [20]. There was no obvious implant loosening in these cases, and the polyethylene liners and femoral heads were exchanged during the revision surgery. Their modular femoral neck components remained well fixed in the head bores, and no clear evidence of fretting, micromotion, corrosive damage, and impingement scars were found. There was no significant difference in the size of pseudotumor between symptomatic and asymptomatic patients (*P* = 0.061). The intraoperative histopathological findings of the pseudotumors revealed foreign body reactions to large polyethylene wear particles with 20–1000 μm, with no histological evidence of corrosion at the head-neck interfaces.

**Table 2:** Demographic data of patients with pseudotumor

Case	Age/ Gender	Bearing	Pseudotumor			Revision	Wear rate (mm/year)	Metal ion level		
			Size (cm^3^)	Type	Symptom			Co (μg/L)	Cr (μg/L)	Co/Cr ratio
1	63/ F	MoP	4.0	I	–	–	0.14	0.4	0.5	0.8
2	43/ F	MoP	20.2	III	+	+	0.14	0.3	0.3	1.0
3	60/ F	MoP	6.2	I	–	–	0.13	0.6	0.5	1.2
4	39/ M	MoP	3.5	I	+	+	0.17	0.3	0.5	0.6
5	54/ F	MoP	0.6	I	–	–	0.09	0.5	0.4	1.3
6	49/ M	MoP	1.1	I	–	–	0.11	0.2	0.2	1.0
7	66/ M	MoP	0.9	I	–	–	0.11	0.4	0.3	1.3
8	52/ F	MoP	3.1	I	+	+	0.21	0.3	0.3	1.0
9	67/ F	MoP	0.9	I	–	–	0.17	1.2	3.3	0.4
10	39/ M	MoP	15.9	I	+	+	0.30	0.5	0.4	1.3
11	52/ F	MoP	4.2	I	–	–	0.13	0.2	0.4	0.5
12	53/F	MoP	10.5	II	–	–	0.19	0.4	0.3	1.3
13	53/ F	CoP	12.9	II	–	–	0.19	0.2	0.2	1.0
14	56/ F	CoP	2.0	I	–	–	0.13	0.8	0.5	1.6
15	57/ F	CoP	13.8	III	–	–	0.13	0.3	0.2	1.5
16	58/ F	CoP	1.6	I	–	–	0.15	0.3	0.3	1.0
17	49/ F	CoP	3.1	I	–	–	0.19	0.2	0.2	1.0
18	51/ M	CoP	6.3	I	–	–	0.12	0.2	0.3	0.7
19	59/ F	CoP	11.4	I	+	–	0.19	0.2	0.5	0.4
20	60/ F	CoP	7.5	I	–	–	0.11	0.3	0.4	0.8
21	41/ F	CoP	1.2	I	–	–	0.16	0.3	0.3	1.0
22	66/ F	CoP	0.8	I	–	–	0.14	0.5	0.4	1.3

*MoP* metal-on-polyethylene; *CoP* ceramic-on-polyethylene; *Co* cobalt; *Cr* chromium

**Fig. 2:**
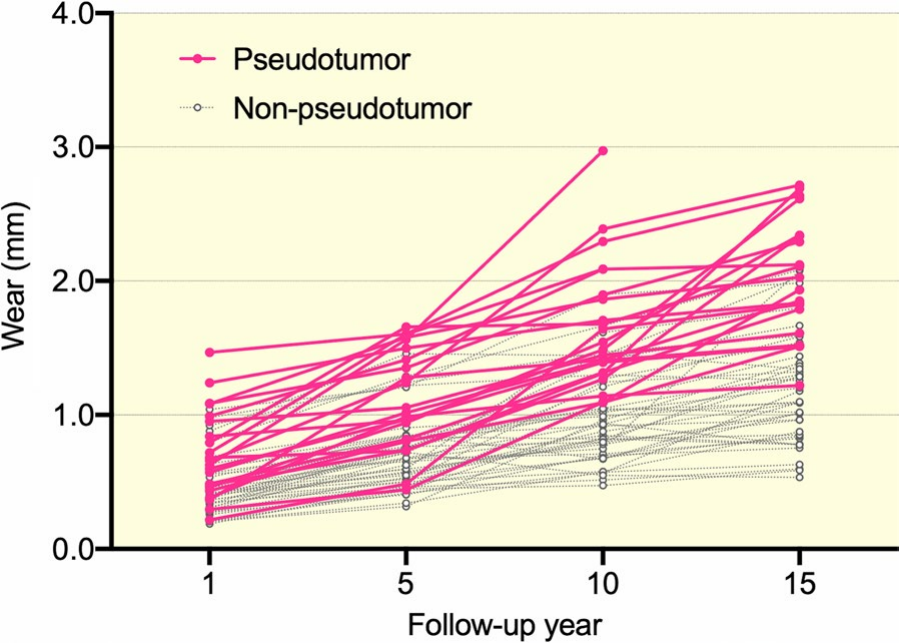
The head penetration plots of patients with pseudotumor (continuous pink lines) and without pseudotumor (dotted grey lines) are shown. Patients with pseudotumors tended to have higher head penetration. The head penetration measurements of individual THAs are connected by a continuous and dotted line

**Fig. 3:**
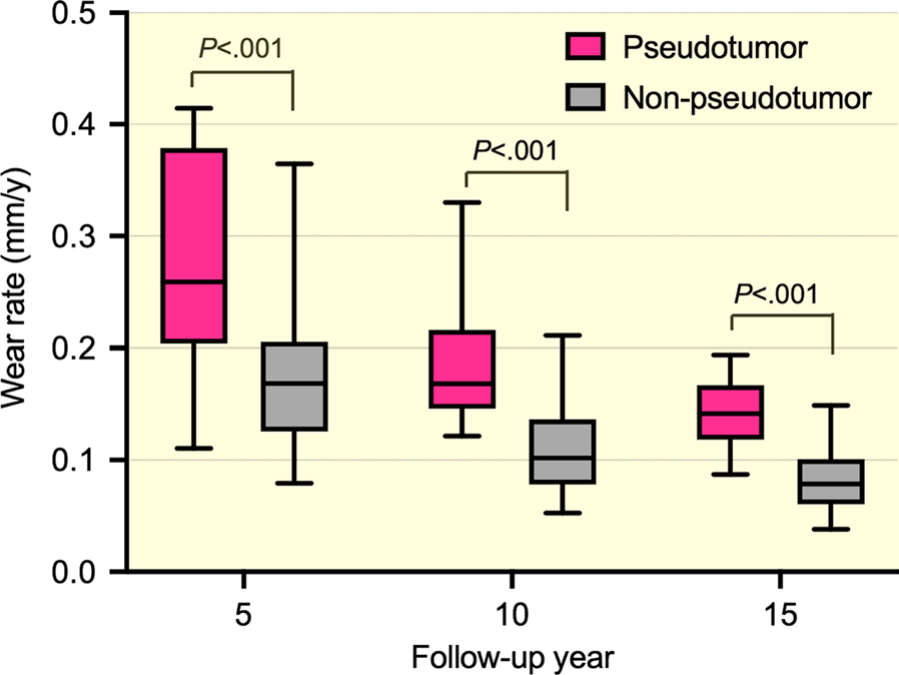
Graph showing a comparison of head penetration rates at 5, 10 and 15 years postoperatively in a patient with and without pseudotumor

Table 1 showed significant differences between patients with and without pseudotumor in age at surgery (*P* = 0.029, *r* = 0.29), preoperative hip extension limitation (as defined by the hip not being able to extend to 0º, or neutral, *P* = 0.033, *V* = 0.27), frequency of acetabular osteolysis (*P* = 0.028, *V* = 0.33), PT at 15 years (*P* = 0.041, *r* = 0.29), changes in cup inclination (*P* = 0.025, *r* = 0.29), change in cup anteversion (*P* = 0.001, *r* = 0.43), and change in PT (*P* = 0.008, *r* = 0.33) between 5 and 15 years. The median (IQR) wear increased over time from 0.46 (0.37–0.59) mm at 1 year to 1.20 (0.85–1.39) mm at 15 years in patients without pseudotumor, while it increased from 0.53 (0.46–0.80) mm at 1 year to 2.02 (1.74–2.34) mm at 15 years in those with pseudotumor (Fig. 2). When compared to the non-pseudotumor group, the pseudotumor group showed significantly more wear at 5 (*P* < 0.001, *r* = 0.49), 10 (*P* < 0.001, *r* = 0.66), and 15 years (*P* < 0.001, *r* = 0.66) (Table 3). Moreover, the median (IQR) wear rates evaluated at 5, 10 and 15 years were 0.17 (0.13–0.20), 0.10 (0.08–0.13), and 0.08 (0.06–0.10) mm/years in the non-pseudotumor group, while were 0.26 (0.21–0.37), 0.17 (0.15–0.21) and 0.14 (0.12–0.17) mm/years in the pseudotumor group (Table 3 and Fig. 3). The wear rates in the pseudotumor patients were always significantly higher than in the non-pseudotumor patients (*P* < 0.001, *r* > 0.5 for all comparisons). On the other hand, no significances between the groups were found regarding body mass index (BMI), head materials, cup diameter, polyethylene liner thickness, HHS, cup orientation at 5 and 15 years, frequency of femoral osteolysis and metal ion levels (Table 1).

**Table 3:** Comparison of polyethylene wear diagnostic measures between patients with and without pseudotumor

	Non-pseudotumor (n = 42)		Pseudotumor (n = 22)		*P* value	Effect size
	Median	IQR	Median	IQR		
*Wear (mm)*						
1 y	0.46	0.37–0.59	0.53	0.46–0.80	0.06	n/a
5 y	0.80	0.57–0.90	1.23	0.96–1.48	** < 0.001**	0.49
10 y	1.00	0.71–1.21	1.67	1.41–1.89	** < 0.001**	0.66
15 y	1.20	0.85–1.39	2.02	1.74–2.34	** < 0.001**	0.66
*Wear rate (mm/y)*						
5 y	0.17	0.13–0.20	0.26	0.21–0.37	** < 0.001**	0.53
10 y	0.10	0.08–0.13	0.17	0.15–0.21	** < 0.001**	0.64
15 y	0.08	0.06–0.10	0.14	0.12–0.17	** < 0.001**	0.65

Numbers in bold are statistically significant

*IQR* interquartile range; *n/a* not available; *r* effect size

### ROC analyses for prediction of pseudotumor formation

**Fig. 4:**
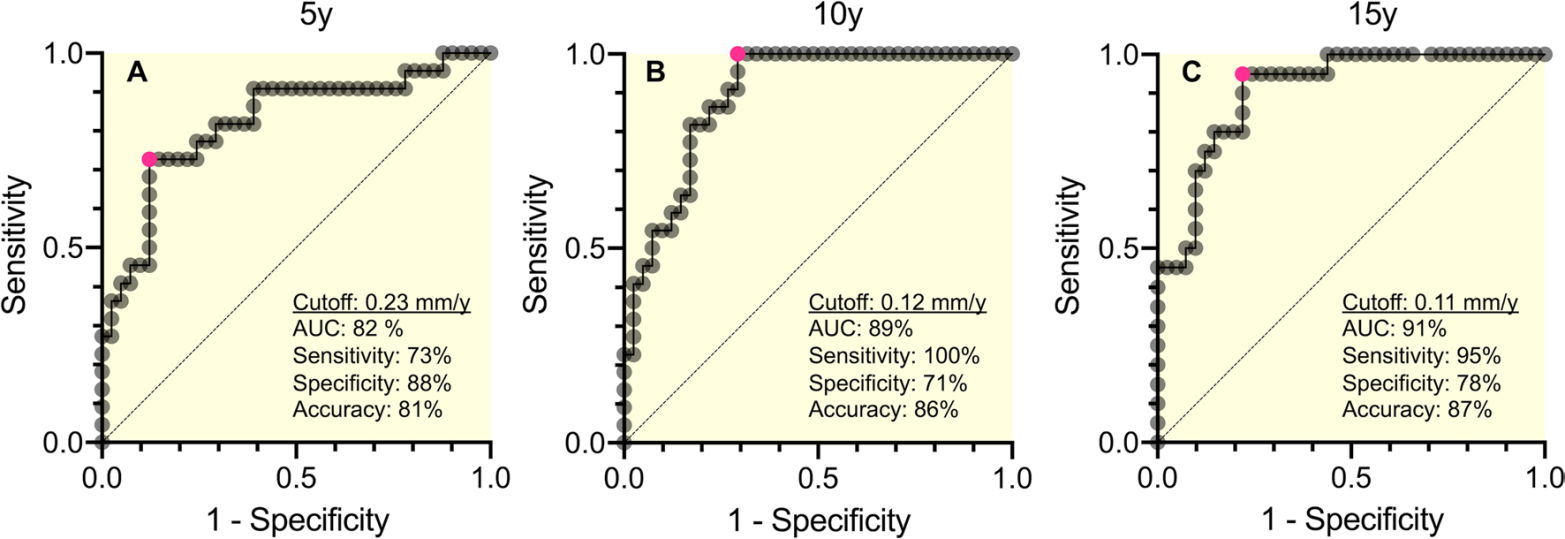
The figures show the receiver-operator characteristic (ROC) curves at 5 (A), 10 (B) and 15 (C)-year postoperative follow-up. The optimal cutoff values are represented by pink circle markers

Figure 4A–C depicts the ROC curves (*i.e.*, the plot of sensitivity vs. 1–specifity) of wear rates determined at 5, 10 and 15 years with AUC values of 82, 89 and 91%, respectively. The most sensitive and specific cutoff values of wear rates at 5, 10 and 15 years were 0.23 (sensitivity [*Se.*] = 73%; specificity [*Sp.*] = 88%; accuracy [*Ac.*] = 81%), 0.12 (*Se.* = 100%; *Sp.* = 71%; *Ac.* = 86%), and 0.11 mm/y (*Se.* = 95%; *Sp.* = 78%; *Ac.* = 87%), respectively.

## Discussion

The most common failure mode related to tribocorrosion in hard-on-soft THA is mechanically-assisted crevice corrosion (MACC), which has been observed particularly when a large diameter head was used [21–24]. In modular MoP THA, the prevalence of MACC has been reported in the range of 1.1–4.7% [21–24]. Considering these percentages, the primary mechanism of pseudotumor in our cases apparently may not be associated with MACC because of much higher incidence of pseudotumor (34%) despite the cohorts including both MoP and CoP using small femoral heads. Blood/serum metal ion concentrations have been shown to be reliable predictors of ALTR due to head-neck taper corrosion [25, 26]. Fillingham et al. [25] showed very high accuracy (AUC = 99%, *Se.* = 100%, and *Sp.* = 90%) in diagnosing ALTR associated with corrosion in MoP THA when the optimal cutoff of Co ion level was set at 1.0 μg/L. Kwon et al. [26] suggested that a threshold serum Co level of > 1.0 μg/L and a Co/Cr ratio of > 2.0 μg/L are predictive indicators of ALTR after MoP THA. In our cases, only one patient with pseudotumor (Case 9 in Table 2) had a Co ion level exceeding 1.0 μg/L, but none of the patients exceeded the Co/Cr ratio of 2.0 μg/L at the time of follow-up. Although the influence of metal wear and ions might not be eliminated completely, no significance was noted in the Co/Cr ratio between the two cohorts (Table 1), which could be a more sensitive predictor for ALTR than serum Co and Cr levels alone [27]. In the 15-year follow-up, the pseudotumor group had 1.8 times greater median linear wear rate than the non-pseudotumor group (0.14 vs. 0.08 mm/year, *P* < 0.001, *r* = 0.65, Table 3). Excessive polyethylene wear was identified as the primary factor in the causation of granulomatous reactions by Santavirta et al. [28] and Austin and Stoney [9]. Howie et al. [10] also identified excessive polyethylene wear as the cause of bursal masses. We recently reported 2 cases of pseudotumor following MoP THA, and histological examination revealed a foreign body reaction to polyethylene particles with no adverse reaction to metal debris and no severe signs of corrosion at the head-neck junction [8]. Therefore, polyethylene wear particles might possibly contribute to the development of ALTR in our series.

It is important to note that factors associated with an increased/decreased polyethylene wear rate include patient-related factors, surgeon-related factors, and implant-related factors. Furthermore, common wear-related factors have been identified as gender, age, BMI, activity level, head diameter, head material, liner thickness, cup diameter, cup alignment and pelvic change [2, 3, 29, 30]. In the present study, we found significant differences in some wear-related factors between the 2 patient groups with and without pseudotumor (Table 1). In the 5–15 years follow-up period, the pseudotumor group had primary THA at a significantly younger age and showed a significant increase in cup inclination, anteversion and PT (more retroverted pelvis) than the non-pseudotumor group. Note that the percentage of the patients with preoperative hip extension limitation was significantly higher in the pseudotumor group than in the non-pseudotumor group (41% vs. 17%, *P* = 0.033, Table 1). Some authors pointed out that there may be a relationship between postoperative increase in PT and pre-existing flexion contracture of the hip [31–33]. In one study, Sakaguchi and Suenaga [31] followed 45 patients who underwent THA and showed that the degree of preoperative hip extension limitation was correlated with the postoperative increase in PT due to surgical release of the contraction of the anterior hip capsule. Nevertheless, change in PT angle should be multifactorial including numerous patient factors (*e.g*., age, gender, vertebral fractures, preoperative anterior PT, spinal degeneration, and history of spinal fusion surgery) [34]. A detailed analysis of these factors in relation to the development of polyethylene wear and pseudotumor is beyond the scope of this work and will be subject of future studies.

The cellular response to polyethylene wear debris might be affected not only by the number of particles but also their size, morphology, and distribution. The cellular response to different sizes of polyethylene wear particles has previously been studied. Howie et al. [10] demonstrated using rat knees that the multinucleate giant-cell response occurred in response to both large polyethylene particles and aggregates of small polyethylene particles. In general, macrophages are capable of phagocytosing very small particles (< 5 μm), whereas larger particle sizes (> 10 μm) induce the formation of foreign body giant cells [35]. As a result, when wear particles of a size that macrophages cannot phagocytose are generated, a foreign body giant cell-based reaction may occur [12], potentially promoting pseudotumor formation in the periprosthetic soft tissue. In our cases, all 4 patients receiving revision THA had a high wear rate of 0.14–0.30 mm/y (Table 2) and large polyethylene wear particles with a size of > 20 μm, with 2 of the 4 patients having supra-macroparticles of > 100 μm [8]. Thus, the long-term accumulation of wear and the presence of large polyethylene particles might have a role in the pseudotumor formation by stimulating a giant cell-based reaction.

Large polyethylene particles are generated commonly because of abnormal wear attributed to excessive mechanical loading such as dislocation, subluxation, impingement, and edge loading [8, 36]. The significant change in acetabular cup orientation can become a risk factor for such abnormal wear modes. As mentioned, significantly greater increases in cup inclination and anteversion were noted postoperatively in the pseudotumor group compared to the non-pseudotumor group (Table 1). In this circumstance, excessive mechanical loading associated with subluxation, impingement, and /or edge loading seems more likely to occur in the pseudotumor group than the non-pseudotumor group. Babisch et al. [37] reported that PT can have the dramatic influence on cup orientation. They showed that anteversion changed approximately 4° for every 5° of change in the PT angle, whereas inclination was less affected, changing approximately 1.5° for every 5° of change in the PT angle. The increases in anteversion (5.4° and 2.9°) and inclination (3.3° and 2.0°) observed in the pseudotumor and non-pseudotumor groups were most likely due to the increases in PT angles between 5 and 15 years (7.2° and 4.1°) (Table 1).

At the mean follow-up of 16.9 years, the incidence of pseudotumor formation in our patients was 34% (22/64 hips). Although there was no significant difference in its incidence between the head types, 35% (12/34 hips) of MoP patients and 33% (10/30 hips) of CoP patients experienced the development of pseudotumor (*P* > 0.999) (Table 1). At 5–15 years, the pseudotumor group had 1.5–1.7 times greater total wear than the non-pseudotumor group (*P* < 0.001 for all, Table 3). According to an analysis of the ROC curves in Fig. 4, the cutoff level of the wear rate to optimally distinguish between pseudotumor and non-pseudotumor patients was 0.11 mm/year at 15 years, demonstrating a good discriminative ability (AUC = 91%; *Se.* = 95%; *Sp*. = 78%; *Ac.* = 87%). This wear threshold for pseudotumor is higher than that to increase the frequency of osteolysis reported previously in the range of 0.05 [38] to 0.1 mm/year [2, 3]. Some authors [39–42] hypothesized that more than a foreign body reaction is necessary to produce granulomatous pseudotumors and movement or loosening is needed to stimulate the reaction in the presence of wear debris. They suggested that osteolysis and/or mechanical movement of prosthesis may tend to appear before the development of pseudotumor. In our patients, the development of all pseudotumor was found only around acetabular components. Although no clear radiographic evidence of loosening was found, the pseudotumor group showed a significantly higher incidence of acetabular osteolysis than the non-pseudotumor group (41% vs. 14%, *P* = 0.028). In the above contexts, acetabular osteolysis appears to induce the development of pseudotumor.

This study had some limitations. First, it was retrospective study with a relatively small sample size and the two cohorts were not randomized. However, large effect sizes were obtained for all significant comparisons in the wear rates, suggesting a strong degree of practical significance for the primary results (Table 3). Therefore, we considered our sample size (64 hips) to be appropriate for this study although a larger sample size might be more helpful in detecting the statistical significances for the secondary variables. Second, we were unable to determine the precise time of onset of pseudotumor because MARS-MRI was not routinely performed on patients without asymptomatic pseudotumor prior to the final follow-up of 15 years. Third, the intraoperative histopathological analysis was performed for only 5 of 22 pseudotumor patients and their periprosthetic masses were identified as foreign-body granuloma formation triggered by excessive wear of polyethylene component. However, since the remaining 17 patients was not diagnosed histologically, we termed all masses detected on MARS-MRI as pseudotumors in this study. Fourth, the evaluation of the two-dimensional polyethylene liner wear (femoral head penetration) on AP-pelvic radiographs taken in the supine position using the Martell´s Hip Analysis Suite software has some limitations in accuracy compared to the gold standard of Radiostereometric analysis (RSA) as a method for measuring three-dimensional relative displacements. However, since we studied a 30-kGy cross-linked polyethylene showing a relatively high linear wear, its detection can be accomplished with an acceptable accuracy. Bragdon et al. [43] previously found good agreement between RSA and Martell measurements when the steady-state wear rates were compared, which is the most important parameter in the long-term performance of the component. Fifth, head penetration was evaluated on radiographs taken in a supine position. However, we used a method introduced by Moore et al. [44], in which the lower limbs were internally rotated. They showed that the two-dimensional results of head penetration did not change substantially between radiographs made with the patient standing and bearing weight with the lower limbs in neutral rotation and those with the patient supine with the lower limbs internally rotated, most likely because the hip capsule and soft tissues surrounding the implant were strained enough to keep the head in nearly the same position regardless of whether the hip was loaded or unloaded. Although, in this original publication [44], only a small study group (37 patients) was investigated, we adopted their methods in a supine position because the standing position seemed more likely to account for a higher degree of individual variability in the radiographic analysis due to an age-related pelvic retroversion in the long-term follow-up. Finally, care should be taken in generalizing the results to other types of UHMWPE, especially those irradiated with greater than sterilization dose (25–40 kGy) and stabilized with antioxidants because of possible differences in size and shape distribution of wear particles and their biological activities [8, 45]. In fact, UHMWPE components studied are not commonly used in contemporary THA. However, there are currently some opportunities to examine/revise patients who have been implanted with the similar types of conventional polyethylene for a few decades.

Despite the aforementioned limitations, our results might provide new insights into excessive polyethylene wear potentially leading to the future development of both pseudotumor and osteolysis. Further studies are needed to clarify the direct relationship between polyethylene wear and pseudotumor and the mutual effects of osteolysis and pseudotumor in particle reactions.

## Data Availability

The data presented in this study are available from the corresponding author.

## Abbreviations

ALTR: Adverse local tissue reactions
THA: Total hip arthroplasty
Co: Cobalt
Cr: Chromium
UHMWPE: Ultra-high molecular weight polyethylene
MoP: Metal-on-polyethylene
HHS: Harris hip score
AP: Anteroposterior
PT: Pelvic tilt
SFP: Sacro-femoral-pubic
ROC: Receiver operating characteristic
AUC: Area under the curve
BMI: Body mass index
MACC: Mechanically-assisted crevice corrosion
RSA: Radiostereometric analysis
